# Ovarian Leydig Cell Tumor and Ovarian Hyperthecosis in a Postmenopausal Woman: A Case Report and Literature Review

**DOI:** 10.3390/medicina59061097

**Published:** 2023-06-06

**Authors:** Diana Bužinskienė, Rūta Marčiukaitytė, Evelina Šidlovska, Vilius Rudaitis

**Affiliations:** 1 Faculty of Medicine, Vilnius University, LT-03101 Vilnius, Lithuania; 2 Center of Obstetrics and Gynecology, Institute of Clinical Medicine, Faculty of Medicine, Vilnius University, LT-08661 Vilnius, Lithuania; 3 National Center of Pathology, Affiliate of Vilnius University Hospital Santaros Klinikos, LT-08406 Vilnius, Lithuania

**Keywords:** ovarian Leydig cell tumor, ovarian hyperthecosis, hyperandrogenism, postmenopausal women, salpingo-oophorectomy

## Abstract

Ovarian Leydig cell tumor is a rare type of ovarian steroid cell neoplasms, presenting in only 0.1% of all ovarian tumor cases, and is generally androgen-secreting and unilateral. Although they are often malignant non-spreading tumors, which have excellent prognosis, benign ovarian Leydig cell tumors with low-risk malignancy can be also detected. Ovarian hyperthecosis is a rare non-neoplastic disorder, in most cases bilateral. Ovarian tumors and ovarian hyperthecosis are one of the main causes of hyperandrogenism in postmenopausal women, a condition strongly associated with both hormonal and metabolic changes. Here, we report a 65-year-old patient with complaints of excessive body hairiness and alopecia. The laboratory investigation showed increased levels of serum testosterone and dehydroepiandrosterone sulfate (DHEA-S). Imaging, including transvaginal ultrasound and pelvic MRI revealed the presence of two masses in the ovaries. The patient underwent a laparoscopic bilateral salpingo-oophorectomy due to the ovarian tumors unknown etiology, and histopathological examination revealed a unilateral benign left ovarian Leydig cell tumor with bilateral ovarian stromal hyperplasia and ovarian hyperthecosis. Making differential diagnosis between ovarian tumors and ovarian hyperthecosis is difficult. Bilateral salpingo-oophorectomy is the treatment of choice in postmenopausal women with benign Leydig cell ovarian tumor, as well as ovarian hyperthecosis, as it offers both a cure and diagnostic confirmation.

## 1. Introduction

After menopause, the ovaries keep secreting sex hormones in small amounts. Oestrogen levels decline abruptly, while androgen secretion decreases more gradually. Luteinizing hormone stimulation maintains ovarian androgen secretion in postmenopause [1,2]. Despite the physiological hormone processes in menopause, the presence of true virilisation characteristics such as alopecia, hirsutism, acne, deepening of the voice, or clitoromegaly must be analysed cautiously, as these disorders can be caused by various non-tumorous or tumorous conditions with androgen hyperproduction [1,2,3,4]. Identifying the precise cause of hyperandrogenism can be a complex task, requiring a comprehensive approach that combines clinical assessment, laboratory testing, imaging techniques and, in certain cases, postoperative histopathological examination. Ovarian hyperthecosis (OH) is one of the functional (non-tumorous) disorders that cause hyperandrogenism. In OH, ovarian stromal hyperplasia with various nests of luteinized thecal cells can be observed. These cells differentiate from ovarian interstitial cells and produce an excessive amount of testosterone. The etiopathogenesis of this disorder remains unclear. However, there are studies, which suggest genetic predisposition [5,6]. Leydig cell tumors are rare, accounting for approximately 0.1% of all ovarian tumors. Their natural history, management approach, and prognosis can be challenging to understand due to their rarity and limited research on them. Typically, they are androgen-secreting, unilateral, and can be either benign or malignant. Malignant non-spreading tumors occur more frequently and have an excellent prognosis [4]. Tumorous Leydig cells have high steroidogenic activity leading to increased levels of testosterone. Postmenopausal hirsutism and virilisation appear in 75% of all Leydig cell tumor cases. According to the literature, the mean time between the presence of symptoms and histological diagnosis of Leydig cell ovarian tumor is approximately five years [4,7]. This article describes a rare case of unilateral benign Leydig cell ovarian tumor combined with bilateral ovarian stromal hyperplasia and OH in a postmenopausal woman. 

## 2. Case Report

**Figure 1:**
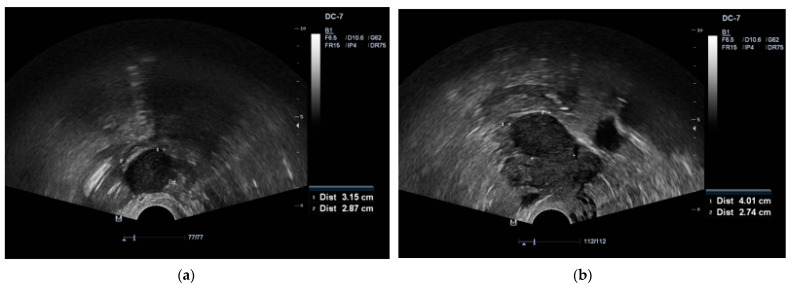
(**a**) Transvaginal ultrasound of a 3.1 × 2.8 cm lesion in the right ovary. (**b**) Transvaginal ultrasound of a 4.0 × 2.7 cm lesion in the left ovary.

A 65-year-old Caucasian female (parity 2, abortions 1, and miscarriages 0) presented to an endocrinologist due to excessive body hairiness and alopecia. Over the past year she experienced these symptoms, which caused the patient significant psychological distress. The patient experienced menopause at age 55 years. She had a history of hysteroscopic polypectomy and other concomitant diseases, including primary hypertension, neurosensorial hearing loss, non-toxic multinodular goiter, obesity, detected multiple cystic lesions in the pancreas, characteristic of intraductal papillary mucinous neoplasm (IPMN), as well as bilateral parapelvic renal cysts. A Pap smear was performed several years ago, but the patient did not know the results. Laboratory testing showed increased levels of serum testosterone and dehydroepiandrosterone sulfate: total testosterone 25.96 nmol/L (normal range, 0.43–1.24), dehydroepiandrosterone sulfate (DHEA-S) 6.25 µmol/L (normal range, 0.8–4.9). Transvaginal ultrasound revealed the presence of two tumors in both ovaries, with the right measuring 3.1 × 2.8 cm and the left measuring 4.0 × 2.7 cm (Figure 1a,b).

**Figure 2:**
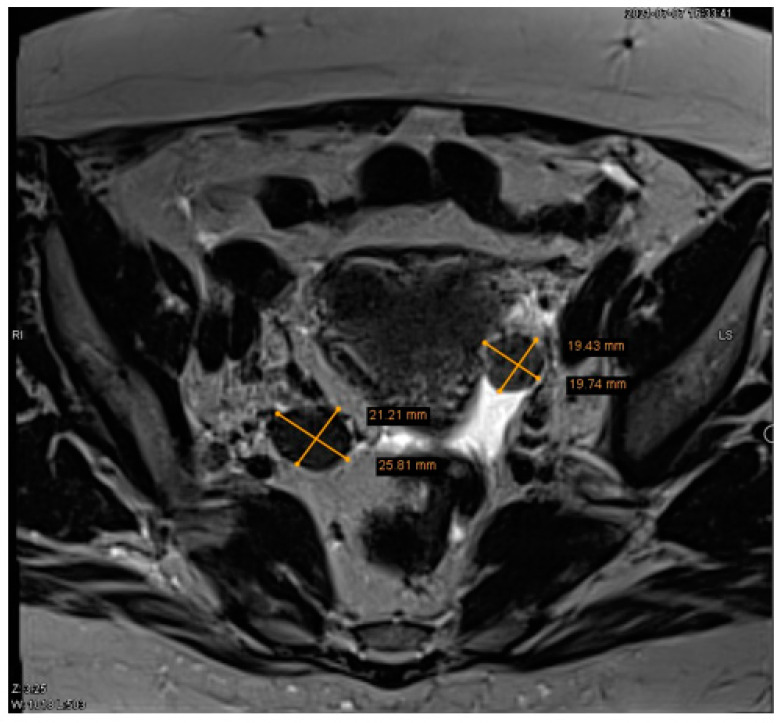
Pelvic magnetic resonance imaging (MRI) of two ovarian lesions. The pelvic MRI revealed a circumscribed left ovarian structure measuring 1.9 × 1.9 cm and a circumscribed right ovarian structure measuring 2.1 × 2.6 cm.

Subsequently, the patient was referred to the gynaecology department due to suspected ovarian tumors of unknown aetiology. Ovarian tumor markers were conducted for the patient and they were within normal ranges (cancer antigen 125 (CA-125) 25.5 kU/L (normal range, <35) and human epididymis 4 (HE4) 31.6 pmol/L (normal range, <140)). A pelvic MRI confirmed the presence of two low-intensity masses in ovaries bilateral, with the left mass measuring 1.9 × 1.9 cm and the right mass measuring 2.1 × 2.6 cm (Figure 2). Additionally, a small amount of free fluid in the pelvis was observed.

**Figure 3:**
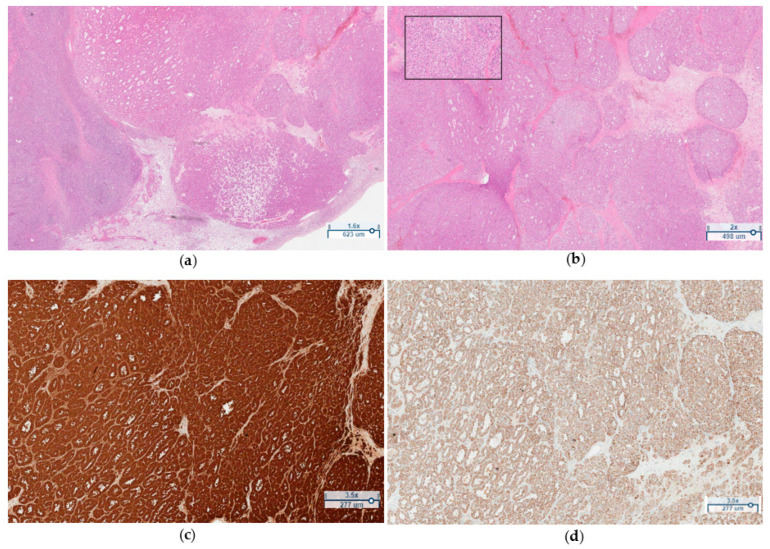
(**a**) Ovarian stroma (on the left) and a well defined Leydig cell tumor (on the right) (H&E, 1.6×). (**b**) Higher magnification showing polygonal cells with fine granular to vacuolated cytoplasm, round to oval nuclei, and visible nucleoli (H&E, 2×). (**c**) Positive staining of tumor cells for calretenin (Calretenin, 3.5×). (**d**) Positive staining of tumor cells for melan A (MelanA, 3.5×).

The patient underwent laparoscopic bilateral salpingo-oophorectomy. During the procedure, pelvic washing was performed by spilling a 50 mL solution of 0.9% NaCl onto the surface of the uterus, ovaries, and pelvic peritoneum. A portion of the solution was aspirated and sent for cytological evaluation, but no tumorous cells were detected. The tumor was removed from the pelvic cavity without rupture, and the contents of the tumor did not enter the peritoneal cavity. Histopathological examination confirmed the presence of a well-defined benign Leydig cell tumor measuring 1.5 × 1.5 × 1.5 cm in the left ovary. The tumor exhibited nests, trabeculae, or a pseudotubular pattern separated by collagenous septa of varying widths. The tumor cells were polygonal in shape, with cytoplasm ranging from fine granular to vacuolated. Their nuclei were round to oval with visible nucleoli. Some cells contained intranuclear eosinophilic inclusions, and no definitive Reinke crystals were identified. There was 1 mitosis observed per 10 HPF. Immunohistochemical stains showed positivity for calretenin, inhibin, MelanA, while the stain for WT-1 was negative (Figure 3a–d). In both ovaries, hyperplastic stroma with luteinized cells, indicative of ovarian hyperthecosis, was observed. 

After the surgical procedure, the patient’s general condition was satisfactory. Postoperative recovery was uneventful. Unfortunately, the patient did not have further contact with the department after the surgery, thus follow-up was not possible.

## 3. Materials and Methods

All tumor slides (eight tumor slides were performed) were stained with hematoxylin and eosin. For immunohistochemical analysis, paraffin-embedded sections cut at 2 µm were deparaffinized and rehydrated, and endogenous peroxidase activity was blocked using 3% hydrogen peroxidase. Immunohistochemistry was performed using the monoclonal antibodies against inhibin, calretinin and Melan A. Scoring was based on the percentage of positive tumor cells and the intensity of positivity. Proper positive and negative controls were included in the analysis.

## 4. Discussion

Making accurate diagnosis of virilisation and hyperandrogenism in postmenopausal women can be challenging due to the diverse range of causes, which consist of non-tumorous and tumorous conditions [1]. Our clinical case shows simultaneous presence of a unilateral benign Leydig cell ovarian tumor and bilateral ovarian hyperthecosis. The initial diagnosis of bilateral tumors of unknown aetiology was based on radiological findings and abnormal laboratory results, and the definitive diagnosis was confirmed postoperatively by histopathological examination.

Ovarian tumors and OH are among the most common causes of hyperandrogenism in postmenopausal women. In clinical practice, there is still a lack of clinical or diagnostic parameters that can reliably differentiate between ovarian tumors and OH. However, certain tendencies and characteristics can be described. Initial evaluation of postmenopausal hyperandrogenism is based on virilising symptoms and their progression. Virilising symptoms, including hirsutism, alopecia, and clitoromegaly are frequently observed in both conditions. However, certain studies indicate that postmenopausal women with ovarian tumors may exhibit a higher prevalence of muscle hypertrophy and deepening of the voice. Typically, the symptoms associated with OH develop gradually over several years, while neoplastic processes tend to have a rapid onset within several months [5]. However, it is difficult to accurately differentiate between non-tumorous and tumorous causes based solely on clinical manifestations. In terms of laboratory findings, high serum testosterone concentration (>5 nmol/L) is seen both in OH and ovarian Leydig cell tumor. Additionally, in postmenopausal women with Leydig cell tumor testosterone levels tend to range from approximately 3 to 25 times above the upper limit of normal. Elevated levels of DHEA-S make tumorous hyperandrogenism more likely, as DHEA-S levels are typically within normal range in ovarian hyperthecosis [4,6]. Transvaginal ultrasound followed by MRI can effectively localize the tumor. Leydig cell tumors usually appear unilateral, are small (1–3 cm), exhibit a solid echotexture in ultrasound and show a hypointense signal on T1-weighted MRI sequences with enhancement observed after contrast administration. Conversely, OH usually affects both ovaries, leading to an increase in their size and volume, with a hypointense signal on T1 or T2-weighted sequences in MRI without contrast-induced enhancement [6]. Due to the difficulties in making accurate differential diagnosis between androgen-secreting ovarian tumors and OH, the final diagnosis, as in our case, is confirmed by histopathological examination [6,8]. The standard treatment of both conditions is bilateral oophorectomy [6]. In our patient, the fallopian tubes of both ovaries were also removed due to the detection of tumorous Leydig cells in the fimbriae and a few cysts in the wall of the left fallopian tube.

OH mostly affects postmenopausal women, and is often referred as a severe or extreme form of polycystic ovary syndrome (PCOS) due to similar clinical manifestations and metabolic processes. Aetiology and pathogenesis of OH are not fully understood. However, the disorder has been associated with the stimulation of ovarian stromal cells by elevated levels of gonadotropins in postmenopause, as well as hyperinsulinemia and insulin resistance which contribute to increased production of ovarian androgens [5,6]. Most women with OH have obesity, hyperinsulinemia and insulin resistance. Therefore, women with OH have an increased risk of metabolic conditions such as hypertension and type 2 diabetes [2]. Additionally, there is an increased risk of endometrial hyperplasia, polyps and endometrioid adenocarcinoma due to hyperestrogenism caused by the aromatization of excessive amounts of testosterone to oestrogens [9]. The direct association between OH and ovarian tumors, particularly Leydig cell tumor, to our knowledge, is not yet well understood.

Ovarian sex cord-stromal tumors are rare, accounting for 5–8% of all ovarian tumors, and less than half of them is considered androgen-secreting. These tumors are classified based on their cell of origin, including Leydig cell tumors, Sertoli cell tumors, granulosa cell tumors, ovarian thecomas, and steroid cell tumors, not otherwise specified. Leydig cell tumors are typically unilateral, with presentation peak at 6th decade of age, with good prognosis [1,2,7,10]. Leydig cell tumors can be either non-hilar or hilar type, based on whether the tumor originates from the ovarian parenchyma or hilus, respectively. The hilar type is more frequently encountered. Clinical and pathological characteristics do not differ between non-hilar and hilar type [10,11]. The histopathological examination of our described case and macroscopically detected solid structures in the ovarian hilum supported the diagnosis of a hilar Leydig cell tumor. Previous clinical cases and studies have indicated an association between Leydig cell tumors and hypertension. Excessive secretion of androgens can also lead to obesity, cardiovascular complications, metabolic syndrome and type 2 diabetes in postmenopausal women [12]. In our described clinical case, the patient was diagnosed with obesity and primary hypertension in the same year as virilising symptoms were observed, suggesting a possible association between OH, Leydig cell ovarian tumor and the development of metabolic manifestations.

Bilateral laparoscopic salpingo-oophorectomy is the most common surgical approach for Leydig cell tumors, other androgen-secreting neoplasms, and OH in postmenopausal women, and generally associated with low morbidity and mortality rates. Complications related to these surgeries are relatively rare and the overall outcomes are favourable. Unilateral laparoscopic oophorectomy may be a choice for reproductive women to preserve fertility. In some cases, total hysterectomy may necessary, as there have been reports of an association between Leydig cell tumor and endometrial hyperplasia, postmenopausal vaginal bleeding, and endometrial adenocarcinoma in nearly 30% of cases [4]. Adjuvant therapy is not typically administered for benign Leydig cell tumors. However, for steroid cell tumors that exhibit malignancy features such as pleomorphism, large mass, advanced stage, or non-operable residual disease, adjuvant therapy consisting of either platinum with BEP (bleomycin/etoposide/cisplatin) or taxane-platinum combination should be recommended [13]. In addition to surgery, lifestyle modifications and weight loss should be considered. Interestingly, some studies have shown that after surgical treatment of ovarian tumors, no significant changes may be observed in insulin sensitivity, body mass index (BMI) and lipid levels [14]. Therefore, lifestyle modifications and weight loss can not only help reduce testosterone levels, but also have positive effects on these metabolic measures. Insulin sensitizers may also supress androgens, although their efficacy in postmenopausal women has not been well established [8]. While there are currently no specific surveillance programmes after surgical treatment of Leydig cell tumor, follow-up is necessary. In general, some studies have suggested a potential association between bilateral oophorectomy in postmenopausal women and increased risk of certain health conditions such as coronary heart disease and hip fracture. The removal of both ovaries can lead to hormonal changes and decreased oestrogen levels, which are known to have protective effects on cardiovascular health and bone density. Therefore, it is important to observe and implement preventive measures for these conditions [15]. We generally recommend evaluating the remission of virilising symptoms, performing laboratory tests to assess androgen hormone levels, as well as performing a physical examination three months after surgery. If clinically indicated, a pelvic ultrasound should be undertaken.

## 5. Conclusions

Ovarian tumors and ovarian hyperthecosis are one of the main causes of hyperandrogenism in postmenopausal women. While ovarian Leydig cell tumors and ovarian hyperthecosis share similarities in terms of androgen excess, they differ in their underlying nature, with Leydig cell tumors being neoplastic disorder, typically occurring unilaterally, and ovarian hyperthecosis being a non-neoplastic disorder, typically affecting ovaries bilateral. Differentiating between these two conditions based on clinical symptoms, laboratory and radiological findings is oftentimes challenging. Bilateral salpingo-oophorectomy is considered the standard surgical treatment for postmenopausal women with benign Leydig cell tumours and ovarian hyperthecosis. This surgical procedure not only provides effective treatment, but also confirms final diagnosis.

## Data Availability

Not applicable.
